# 3CDB: a manually curated database of chromosome conformation capture data

**DOI:** 10.1093/database/baw044

**Published:** 2016-04-14

**Authors:** Xiaoxiao Yun, Lili Xia, Bixia Tang, Hui Zhang, Feifei Li, Zhihua Zhang

**Affiliations:** ^1^CAS Key Laboratory of Genome Sciences and Information, Beijing Institute of Genomics, Chinese Academy of Sciences, Beijing, 100101, China; ^2^University of Chinese Academy of Sciences, Beijing, 100049, China

## Abstract

Chromosome conformation capture (3C) is a biochemical technology to analyse contact frequencies between selected genomic sites in a cell population. Its recent genomic variants, e.g. Hi-C/ chromatin interaction analysis by paired-end tag (ChIA-PET), have enabled the study of nuclear organization at an unprecedented level. However, due to the inherent low resolution and ultrahigh cost of Hi-C/ChIA-PET, 3C is still the gold standard for determining interactions between given regulatory DNA elements, such as enhancers and promoters. Therefore, we developed a database of 3C determined functional chromatin interactions (3CDB; http://3cdb.big.ac.cn). To construct 3CDB, we searched PubMed and Google Scholar with carefully designed keyword combinations and retrieved more than 5000 articles from which we manually extracted 3319 interactions in 17 species. Moreover, we proposed a systematic evaluation scheme for data reliability and classified the interactions into four categories. Contact frequencies are not directly comparable as a result of various modified 3C protocols employed among laboratories. Our evaluation scheme provides a plausible solution to this long-standing problem in the field. A user-friendly web interface was designed to assist quick searches in 3CDB. We believe that 3CDB will provide fundamental information for experimental design and phylogenetic analysis, as well as bridge the gap between molecular and systems biologists who must now contend with noisy high-throughput data.

**Database URL:** http://3cdb.big.ac.cn

## Introduction

Gene expression in eukaryotes is tightly regulated by both cis- and trans-regulatory elements (1). It is well recognized that a large portion of genes physically interact with numerous distal cis-regulatory sequences (2– 4), which may be located tens or even hundreds of kilobases away from the target genes (5). Over the past decade, various experimental protocols have been developed to probe the frequency of physical interactions between distal genomic sequences. Nearly all such technologies are based on chromosome conformation capture (3C) technology (6– 8). The data generated from genome-wide, high-throughput variations of 3C, i.e. Hi-C (9) and chromatin interaction analysis by paired-end tag (ChIA-PET) sequencing (10) have revealed nuclear organization at an unprecedented level. For example, genomes are physically separated into two compartments, named A and B, corresponding to active and inactive parts of the genome, respectively (9). Megabase-sized domains associated with topological structures have also been identified in mammals (11, 12). Recently, ultrahigh-resolution mapping has led to the discovery of sub-TADs (13), preexisting interactions (14) and more detailed subcompartments (15).

Although remarkable progress has been made by high-throughput technologies, the traditional 3C experiment is still the gold standard for determining physical interactions between genomic loci. 3C is irreplaceable in the field of chromatin interaction for many reasons. First, the frequency of interactions along nucleosome fiber exponentially decreases, while genomic distance increases (16), also known as linear proximity effect (LPE). LPE has made it difficult to distinguish functional interactions from background signals. Carefully designed normalization methods have been proposed to correct such bias (17); however, the issue still remains a major concern. Second, ultra-deep sequencing is not a practical solution to this issue. While recent ultra-deep Hi-C data in the GM12878 cell line reached kilobase resolution (15), the sequencing depth needed to reach that resolution is not practical for normal lab operations, even though the price drop in sequencing has exceeded the Moore’s law.

In this study, we introduce the 3CDB database (http://3cdb.big.ac.cn), which provides 3C determined functional chromatin interactions. All data in 3CDB were manually retrieved and curated from published traditional 3C experiments. In total, we extracted 1954 interactions in 15 species from >2500 full-text articles. In addition to raw 3C interaction pairs, we developed a systematic evaluation scheme based on data reliability and classified the interactions into four categories. Therefore, we believe that 3CDB will be a rich resource for the research community.

## 3C Technology

**Figure 1.**
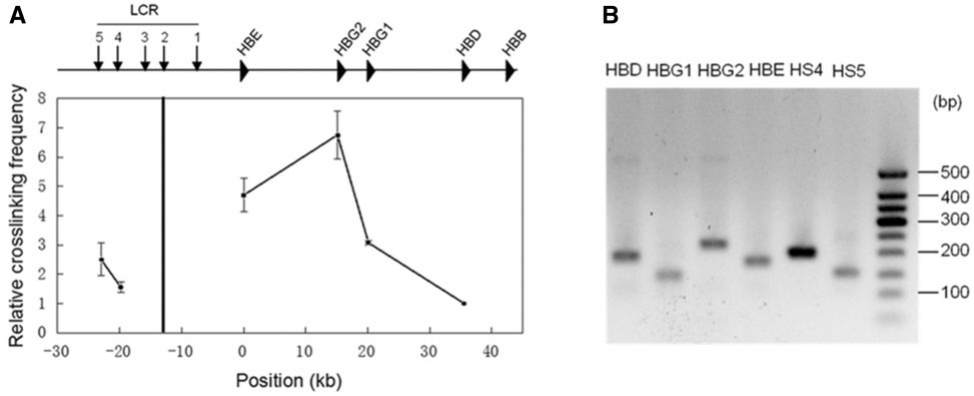
3C analysis of chromatin interactions between the locus control region (HS2) and the rest of the β-globin locus in K562 cells using HindIII restriction enzyme. **A.** The qPCR signals at the tested loci. The black vertical line indicates the anchor. The relative crosslinking frequency was determined by quantitatively comparing ligated genome DNA with the control BAC DNA, and the lowest frequency at the HBD site was set as the unit. The error bar represents the standard error of three independent experiments. **B.** Gel analysis of the qPCR product. All amplicons were proved to be the correct ligated products by Sanger sequencing.

Invented about a decade ago (18), 3C technology has been widely used to study distal regulatory genomic interactions in various organisms. Although the protocols used in laboratories may not identical, the core process always consists of the following steps. The first step employs formaldehyde to cross-link DNA and proteins in intact nuclei. Cross-linked DNA is then subjected to restriction enzyme digestion and ligation at very low DNA concentration, which favors intramolecular ligations over intermolecular ligations. Finally, the relative contact frequency between two loci can be quantified by qPCR using locus-specific primers. To control LPE, a 3C experiment usually examines multiple loci around the target site for a given anchor. To control PCR efficiency, a control template in which all possible ligation products are present in equal abundance is used for comparison with the targeted sample. In Figure 1, we show an example of a 3C experiment at the classical β-globin locus in the K562 cell line.

## 3C Database

### Collection of 3C data

3C data in the literature are presented in different ways, while comparison between loci is critical to identify real interactions. Moreover, since literature mining tools in this complicated situation have performed poorly, we manually searched PubMed and Google Scholar for papers containing keywords related to 3C in their titles or abstracts published from 2002 (Table S1), the year 3C technology was first introduced, to December, 2015. In total, 2635 and 4060 articles were collected from PubMed and Google Scholar, respectively. We noticed that the journals listed in Supplementary Table S2 were the most frequently identified. To further collect articles that may have been missed in the first round, we manually browsed all the journals listed in Supplementary Table S2 published since 2002, and 430 more articles were found. After reading all the abstracts and respective methods, we extracted 3319 data entries. For all data entries, multiple columns are given, including name and genome locus of the genes of interest, DNA sequences and primer strands, species name and genome version, name of cell line, number of experimental repetitions, name of restriction enzyme used and the name and publication date of the citation. In addition, we developed a data evaluation scheme to assess the reliability of each interaction and classified all data into four categories. The details of this data evaluation scheme can be found below.

### Statistics of 3CDB

**Figure 2.**
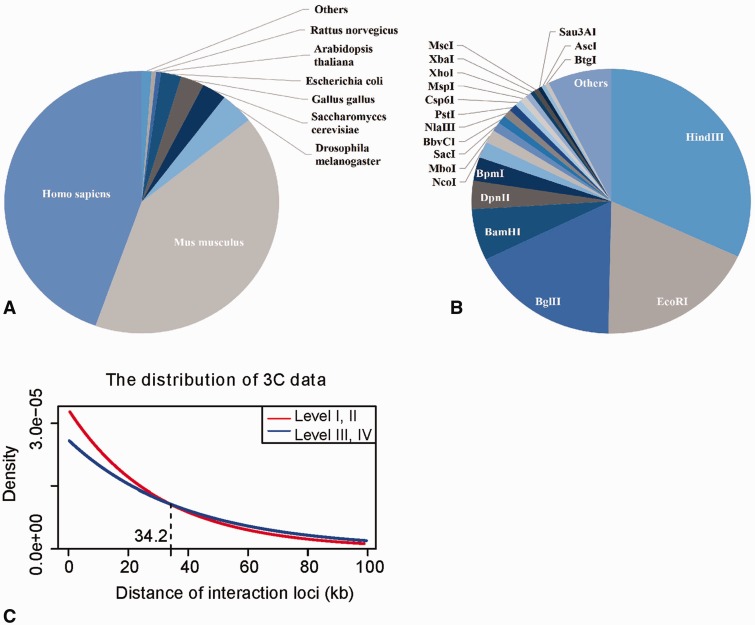
Data statistics. **A.** The distribution of 3C data examined in the species; **B.** The distribution between restriction enzymes used in 3C experiments; **C**. The distribution of 3C measured chromatin interactions over anchor-target distance in the gel image group (red) and the numeric peak group (blue). We fitted 3C data with exponential distribution by R function dexp(). The parameter λ of exponential distribution for the fitted red and blue curve is 3.326e-5 and 2.600e-5, respectively. The Y-axis shows the probability density of chromatin interactions in 3CDB.

The first version of 3CDB, as of December 2015, contains 3319 pairs of chromatin interactions distributed among 17 species and 308 cell lines. With 1479 and 1367 data points, human and mouse, respectively, have become the most widely studied species by 3C technology. A total of 73 restriction enzymes, or enzyme combinations, have been applied in all 3C experiments in the 3CDB. Hind III, Bgl II and EcoR I are the most frequently used restriction enzymes. The distribution of data in species and restriction enzyme types is shown in Figure 2. By our data evaluation scheme, 524 and 669 interactions are in classes I and II, respectively, as defined by gel image. A total of 1266 and 341 interactions retrieved from numeric peaks are in classes III and IV, respectively. Only 519 interactions (519 of 3319, 15.6%) do not have sufficient information with which to define their reliability; thus, the completeness of the scheme in 3CDB is acceptable.

### Web interface

**Figure 3.**
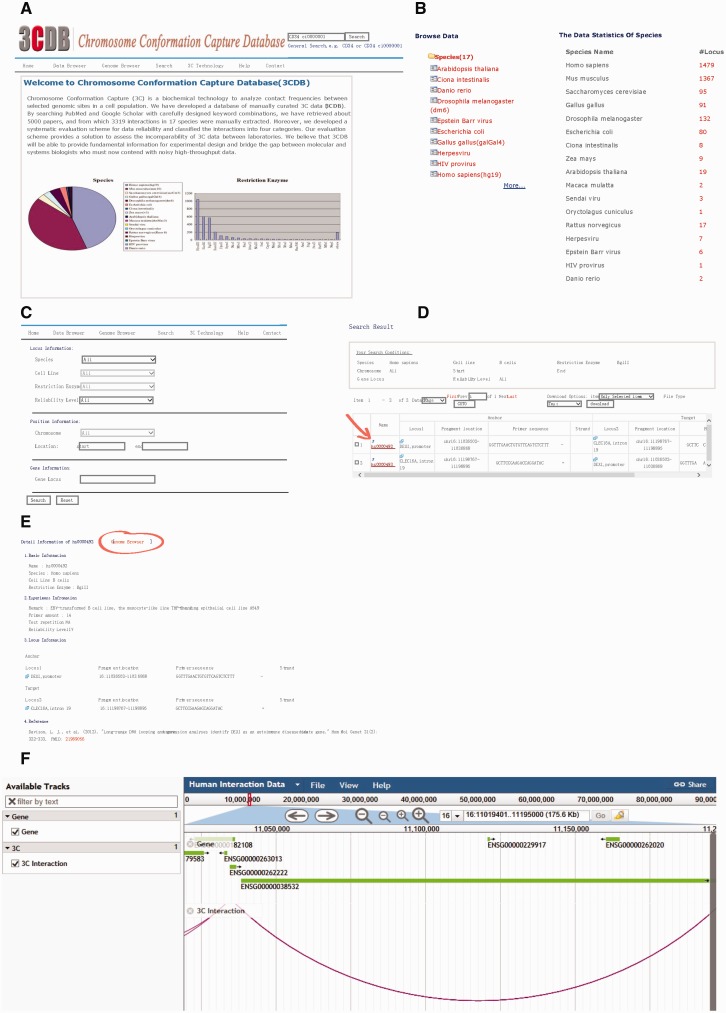
Screen shot of 3CDB contents. **A.** Home Page **B.** Data browser page; **C.** Search Page; **D.** Example of search result; **E.** Example of detailed 3C data. The Genome Browser portal is highlighted (the circle and arrow). **F.** Example of Genome Browser view. The two interaction loci are connected by an arc and gene annotation was represented as horizontal bars.

The 3C database comprises seven pages, including home page, Data browser page, Genome Browser, search page, 3C technology page, Help page and contact page (Figure 3). On the home page, users can find basic information about 3CDB, while the technology and contact pages provide a brief introduction to 3C technology and its variations and contact details, respectively.

The data browser permits users to browse all interactions by species, cell line, or restriction enzyme. Every interaction has a unique interaction ID, which is formatted as the combination of abbreviated species name and an index. Clicking on the interaction ID brings up detailed information about interaction data divided into basic, experimental and locus information, as well as references. Most items are self-explanatory. In the experimental section, a user may find details about the associated 3C experiment, such as number of primers tested around the target locus, number of repetitions, reliability assessment, as defined by our data evaluation scheme (see below), and any additional remarks we think might be useful to users. A simple genome browser has been implemented with an entry in each detailed information page. By clicking on the ‘Genome Browser’, which can also be accessed from the menu bar, the user is taken to a browser showing the locus of the interaction. Interactions are visualized as arcs between loci.

The search page is comprised of three sections: experimental, position and gene information. In the first section, a user can search 3CDB by species, cell line, restriction enzyme, reliability assessment or any combination of the four terms. In the second section, a user can search interactions which have at least one base-pair overlapping with a given genome segment. In the third section, a user can also search 3CDB by gene locus. In fact, the search can be performed with any combination of the terms mentioned above. At the top-right corner, a quick search box is provided, allowing users to perform keyword searches.

The help page provides information necessary for site navigation, as well as an updated FAQ. Users can also download the whole database from the help page.

### 3C data evaluation scheme

3C technology was initially introduced in yeast (18), and it has since been applied to many organisms (8). However, we found considerable variability in the actual 3C experimental protocols established in labs and the way in which authors present their data. These inconsistencies make it difficult to compare 3C results from different experiments, and no valid normalization method is currently available. Therefore, we developed a 3C data evaluation scheme to assess the reliability of each entry in 3CDB and classified data into four categories according to (i) the actual protocols by which the data were generated or (ii) how data were presented (Table 1). Within this classification system, data points within the same category have moderately comparable reliability. These data points were first separated into two large groups: a gel image group and a numeric peak group. By reading the pooled papers, we found that not all articles presented numeric qPCR results. A total of 1193 data points in 3CDB were originally presented as gel image only. Since direct comparison of numeric peaks to such gel images is not well defined, we separated gel data from numeric data. Many such data points, which were retrieved from early publications, report on experiments that only have a single primer designed at the targeted locus and only use ligation-free samples, a bacterial artificial chromosome (BAC), or similar controls. However, the frequency of interactions along the nucleosome fiber exponentially decreases, while genomic distance increases (16). This phenomenon, also known as the LPE has made it difficult to distinguish functional interactions from background signals. Thus, when anchor-target distances are not sufficiently large, a single qPCR bright gel band may be a false-positive. With this concern in mind, we further separated the gel image group into two classes by the distance between anchors and targets (Table 1). A threshold of 34.2 kb was determined by comparing the distribution of the anchor-target distance in the gel image group to the distribution in the numeric peaks group, as a reference. Since interaction loci, as determined by numeric peaks, have the highest ligation frequency among flanking primers, LPE has less effect. In contrast, the gel image group was found to be highly concentrated at distances less than 34.2 kb (Figure 2C), which implies that the data from image gel group were more biased towards closer genome loci; therefore, compare to the numeric peak groups, when anchor-target distance is <34.2 kb, data from the image gel group may contains more LPE affected data. Within the numeric peaks group, data were retrieved from quantitative comparison of relative qPCR signals among a series of candidate primers flanking the targets. Because only the primer with the highest ligation frequency was retrieved as the interaction target, LPE was effectively eliminated as a major concern. Nonetheless, this group could be further classified into two subgroups according to reliability. We noticed that some publications had performed reverse experiments on the loci they studied. These experiments involved setting the target locus as the new anchor and the anchor locus as new target, followed by performing 3C a second time. Since such reverse experiments further decreased the effects of LPE at the anchor site, reliability of resultant data is increased. Therefore, when taken together, numeric peaks with reverse experiments represent the most reliable interactions in 3CDB, followed by numeric peaks without reverse experiments. In the gel image groups, distal data are less noisy compared with proximate data (Table 1).

**Table 1.** 3C data evaluation scheme

Groups	Class name	Definition	Number of interactions
Gel image	I	*d_g_* < 34.2k	524
	II	*d_g_* > = 34.2k	669
Numeric peak	III	Without reverse experiments	1266
	IV	With reverse experiments	341
	Other	Uncategorized	519

3C experimental data were classified into four categories according to reliability. *d_g_* denotes the genomic distance between an anchor and its targets in a 3C experiment. ‘Other’ includes data points that cannot be classified into categories otherwise indicated by Roman numeral, as a result of insufficient information in the original articles.

## Discussion and future directions

### Comparison to similar databases

To the best of our knowledge, databases have been published about genome three-dimensional structure, 3DGD (http://3dgd.biosino.org) (19), as well as the 4DGenome (http://4dgenome.int-med.uiowa.edu) (20). The 3DGD only focuses on high-throughput Hi-C data in yeast, *Drosophila*, mouse and human. Since 3CDB only focuses on traditional 3C-validated chromatin interaction data, 3DGD is not a direct competitor. The 4DGenome attempted to comprehensively collect chromatin interactions by all current 3C-based technologies, e.g. 3C, 4C, 5C, Hi-C, ChIA-PET and IM-PET. However, compared with 3CDB, the 4DGenome only collected a small fraction of 3C data. In the 4DGenome, 3C data were collected for only 4 species compared with 17 in 3CDB. There are 95, 132, 1367 and 1479 interactions for yeast, *Drosophila*, mouse and human in 3CDB, respectively, while only 15 (about 16% of the 3CDB data amount), 6 (5%), 53 (3%) and 144 (10%) 3C interactions were collected for the four species, respectively, in 4DGenome. Moreover, 4DGenome does not classify the data according to reliability, while data classification is a key feature in 3CDB, resulting in a major distinction between the two databases.

### Future directions

We will continue to collect new 3C data, and we plan to add the following features to 3CDB. First, we will include all qPCR signals designed in the original 3C experiment, not just the peak signal. Currently, we only have anchor-target pairs in classes III and IV; however, flanking qPCR signals of the target site shows specificity of the interaction and carries detailed information about the regulatory structure at the locus. Since no flanking qPCR data were available for a large portion of data from the early years of 3C technology (classes I and II), the current version of 3CDB does not include this information. To solve this problem, we are developing an algorithm to simulate such signal from Hi-C data, which will be included in the next version of 3CDB. In addition, we will integrate DNA sequence and epigenetic data, thus providing a solid dataset for gene expression modeling. Second, we will add a more sophisticated visualization interface for interaction loci. Currently, we have visualized chromatin–chromatin interactions as lines and arcs to indicate the loci involved in the interactions. However, arcs do not provide more information than genomic positions *per se.* The 3CDB team will attempt to develop a visualization tool to show physical 3D spatial context at the interaction site, given that high-throughput Hi-C and ChIA-PET data are available in many species (10, 11, 14, 15, 21, 22) and a number of physical structure modeling algorithms have been published (13, 23– 26). Third, although 3CDB focuses on traditional 3C experiments, the scientific community would be benefited by linking such information with the growing body of high-throughput data, or such cytogenetic techniques as fluorescence *in situ* hybridization or spectral karyotyping. With the data evaluation scheme we developed in this version of 3CDB, we should be able to incorporate 3C-verified high-quality interactions into noisy high-throughput data.

## Supplementary data

Supplementary data are available at *Database* Online.

Supplementary Data
