# Supplementary material for: 3CDB: a manually curated database of chromosome conformation capture data
Source: Database (Oxford). 2016 Apr 14;2016:baw044. doi: 10.1093/database/baw044 (PMC4831724; doi:10.1093/database/baw044)
Supplement: Supplementary Data [file supp_2016_baw044_index.html]

Supplementary Data 

# 3CDB: a manually curated database of chromosome conformation capture data

## Supplementary Data

files

- Supplementary Data - docx file
- Supplementary Data - docx file
