# Supplementary material for: 3CDB: a manually curated database of chromosome conformation capture data
Source: Database (Oxford). 2016 Apr 14;2016:baw044. doi: 10.1093/database/baw044 (PMC4831724; doi:10.1093/database/baw044)
Supplement: Supplementary Data [file supp_baw044_Table_S1.docx]

| **Keyword combinations** |
| --- |
| Chromatin, interact, enhancer |
| Chromatin, interact, promoter |
| Chromatin, interact, loop |
| Chromatin, interaction, looping |
| Chromatin, interaction, loop, enhancer |
| Chromosome, interact, looping |
| Chromosome, interaction, looping |
| Chromosome conformation capture |
| Enhancer, promoter, interact |

Table S1 Keyword combinations used for the literature search..

| **Journals** | |
| --- | --- |
| *Nucleic Acid Research* |  |
| *Proceedings of the National Academy of Sciences of the United States of America* |  |
| *The Journal of Biological Chemistry* |  |
| *Molecular and Cellular Biology* |  |
| *The Journal of Immunology* |  |
| *PLoS ONE* |  |
| *Genome Research* |  |
| *Genome biology* |  |
| *The EMBO Journal* |  |
| *Science* |  |
| *Genes & Development* |  |

**Table S2.** Journals with high frequency of hits. These journals have the highest frequency in keyword searches and were manually browsed in all publications since 2002.
